# Selection of best videos of the year for 2016

**DOI:** 10.1590/S1677-5538.IBJU.2017.02.02

**Published:** 2017

**Authors:** Philippe E Spiess

**Affiliations:** M.D, MS, FACS, FRCS(C). Video Section Editor, International Brazilian of Urology Associate member, Department of GU Oncology Moffitt Cancer. Center Associate Professor, Department of Urology. University of South Florida. Tampa, Florida, USA. E-mail: philippe.spiess@moffitt.org

Dear esteemed colleagues and friends,

As we begin a new year, I would like to take this opportunity to thank each of you for your commitment in supporting our journal and expanding its readership. This past year has been truly exceptional in the quality of submissions we have received within the video section of the *International Brazilian Journal of Urology*. We are committed in publishing the highest quality videos detailing novel surgical techniques and approaches by leading surgical teams from across the world. Similarly, we encourage groups pushing the envelope in terms of how we can continually refine the art of surgery and ultimately improve the outcomes of our patients. In this regard, I am pleased to share with you this year’s selection for best videos of the year for 2016. Many individual criteria were taken into account in making this selection including novelty, superior quality in terms of video depiction and narration, and lastly vides that best depict re-defining surgical approaches to urological diseases. On that note, here are the selections:

**First Prize:**

Robotic ureteroureterostomy for treatment of a proximal ureteral stricture by Andrade HS et al. from the Glickman Urological and Kidney Institute, Cleveland Clinic (Cleveland, Ohio) published in volume 42(5):1041-1042, September-October 2016 (available at: http://www.intbrazjurol.com.br/video-section/andrade_1041_1042) (1). This video is truly a masterful depiction on how minimally invasive robotic surgery can be used to tackle benign ureteral stricture disease. Excellent perioperative surgical outcomes are reported and the authors provide sufficient follow-up of 27 months to insure these favorable outcomes are maintained with adequate follow-up. There is no question this surgical approach and specifically this video can be used by surgeons skilled and interested in conducting such surgical procedures.

**Second Prize:**

Robotic repair of vesicovaginal fistula- initial experience by Jairath A et al. from the Department of Urology, Muljbhai Patel Urological Hospital (Nadiad, India) published in volume 42(1):168-169, January-February 2016 (available at: http://www.intbrazjurol.com.br/video-section/video-library/jairath_168_169) (2). The authors presents a surgical series of 8 vesicovaginal fistulas approached using a robotic assisted laparoscopic transabdominal extravesical approach. The authors report excellent outcomes with no recurrences at short-term follow-up of 3 months.

**Third Prize:**

Retzus-sparing robotic-assisted laparoscopic radical prostatectomy: A step-by-step technique description of this first Brazilian experience by Tobias-Machado M et al. from the Departmento de Urologia, Faculdade de Medicina do ABC, Santo Andre, SP, Brazil and other centers of excellence in Sao Paulo, SP, Brazil published in volume 42(6):1250, November-December 2016 (available at: http://www.intbrazjurol.com.br/video-section/tobias-machado_1250_1250) (3). This surgical video is quite elegant in its demonstration that Retzus sparing RRP is not only feasible and reproducible but can enhance continence recovery following initial catheter removal. This is furthermore an excellent depiction of the wonderful surgical collaborations taking place in Brazil.

I would like to conclude this editorial by once again thanking each of you for the support of the **International Brazilian Journal of Urology**. We are committed in publishing the very best original articles and videos from across the world and similarly will continue to do so in a very timely manner, with a rigorous peer review process by the very best subject leaders. Lastly, I send my very best wishes to you and your families for 2017.

Philippe E Spiess, M.D, MS, FACS, FRCS(C) Video Section Editor, International Brazilian of Urology Associate member, Department of GU Oncology Moffitt Cancer Center Associate Professor, Department of Urology University of South Florida Tampa, Florida, USA E-mail: philippe.spiess@moffitt.org
